# Metal-Free Antibacterial Additives Based on Graphene Materials and Salicylic Acid: From the Bench to Fabric Applications

**DOI:** 10.1021/acsami.1c02330

**Published:** 2021-05-26

**Authors:** Giacomo Biagiotti, Annalisa Salvatore, Gianluca Toniolo, Lucrezia Caselli, Maura Di Vito, Margherita Cacaci, Luca Contiero, Tommaso Gori, Michele Maggini, Maurizio Sanguinetti, Debora Berti, Francesca Bugli, Barbara Richichi, Stefano Cicchi

**Affiliations:** † Department of Chemistry “Ugo Schiff”, Università di Firenze, Via della Lastruccia 3-13, 50019 Sesto Fiorentino, Italy; ‡ INSTM (Consorzio Interuniversitario Nazionale per la Scienza e Tecnologia dei Materiali), Via G. Giusti, 9, 50121 Firenze, Italy; § CSGI (Italian Center for Colloid and Surface Science, Via della Lastruccia 3, 50019 Sesto Fiorentino, Firenze, Italy; ∥ Dipartimento di Scienze Biotecnologiche di Base, Cliniche Intensivologiche e Perioperatorie, Università Cattolica del Sacro Cuore, 00168 Rome, Italy; ⊥ Dipartimento di Scienze e Tecnologie Agro-Alimentari, Università di Bologna, Viale G. Fanin 42, 40127 Bologna, Italy; # Cromology Italia S.p.A., Via IV Novembre, 4, 55016 Z.I. Porcari, Lucca, Italy; ∇ Beste S.p.A., Via Primo Levi, 6, 59022 Colle Cantagallo, Prato, Italy; ○ Dipartimento di Scienze Chimiche, Università degli Studi di Padova, Via Marzolo 1, 35131 Padova, Italy; ◆ Dipartimento di Scienze di Laboratorio e Infettivologiche, Fondazione Policlinico Universitario A. Gemelli IRCCS, 00168 Rome, Italy

**Keywords:** cotton fabrics, graphene, graphene oxide, ball milling, salicylic acid, antibacterial activity, quartz crystal microbalance, Raman

## Abstract

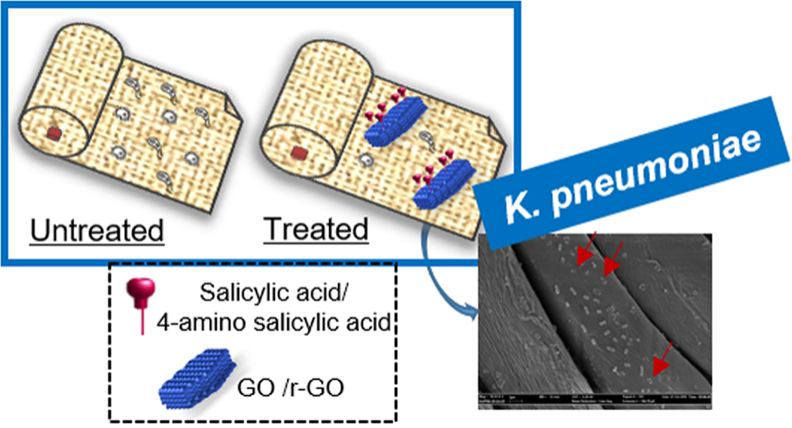

The custom functionalization of a graphene surface allows access to engineered nanomaterials with improved colloidal stability and tailored specific properties, which are available to be employed in a wide range of applications ranging from materials to life science. The high surface area and their intrinsic physical and biological properties make reduced graphene oxide and graphene oxide unique materials for the custom functionalization with bioactive molecules by exploiting different surface chemistries. In this work, preparation (on the gram scale) of reduced graphene oxide and graphene oxide derivatives functionalized with the well-known antibacterial agent salicylic acid is reported. The salicylic acid functionalities offered a stable colloidal dispersion and, in addition, homogeneous absorption on a sample of textile manufacture (i.e., cotton fabrics), as shown by a Raman spectroscopy study, thus providing nanoengineered materials with significant antibacterial activity toward different strains of microorganisms. Surprisingly, graphene surface functionalization also ensured resistance to detergent washing treatments as verified on a model system using the quartz crystal microbalance technique. Therefore, our findings paved the way for the development of antibacterial additives for cotton fabrics in the absence of metal components, thus limiting undesirable side effects.

## Introduction

Antimicrobial and high-performance nanoengineered materials have become highly sought after. In this field, researchers mainly deal with the identification of efficient methodologies for the custom functionalization of material surfaces to provide enhanced colloidal stability and peculiar biological properties, with the development of reliable, eco-friendly, and scalable processes.

In this framework, graphene oxide (GO)^1^ and reduced graphene oxide (r-GO)^2^ showed significant antimicrobial properties toward a broad range of pathogens.^3,4^ These materials have also found application to produce antimicrobial composites,^5−8^ sometimes combined with well-known metal-based antimicrobial additives such as silver nanoparticles (AgNPs).^9−11^ However, for the latter, it has been demonstrated that the extensive use of AgNPs induces bacterial resistance limiting their scope as general antibacterial agents at low concentrations, as they are used on fabrics.^12,13^

On the other hand, despite the basis of antimicrobial mechanisms of graphene-based materials still being controversial, some scenarios have been proposed leading to the identification of key insights helpful for the rational design of efficient graphene-based antimicrobial additives and pointing out some of the issues that need to be addressed.^14,15^ The wrapping of bacteria by graphene-based materials causes physicochemical interactions that give rise to perturbations of the bacterial membrane and deterioration of essential biomolecules (i.e., the “nanoscale dewetting” effect induces collapse of the cell membrane).^16^ Similarly, these interactions isolate bacteria from the nutritive environment^14^ and induce reactive oxygen species (ROS)-dependent and ROS-independent oxidative stress.^14^ However, one main limitation in some applications of graphene-based materials as antimicrobial additives is their tendency to form aggregates. Agglomeration weakens their dispersibility and adsorption ability, hence reducing graphene–bacteria physicochemical interactions.^15,17^ In this regard, a close relationship exists between the graphene surface and antimicrobial efficacy; hence, graphene surface chemical functionalization has been proposed as a valuable approach to prevent particle agglomeration and thus increasing antimicrobial activity.^14,18^ In particular, basal plane destruction and the covalent modulation of the graphene surface with oxygen-containing groups impact the antimicrobial activity either by improving adsorption interactions (i.e., with biomolecules and ions) or enhancing ROS production.^15,19^ However, the scale-up of the functionalization process is the main drawback in handling these materials and it limits industrial-driven applications where either significant batches are required or the use of organic solvents is avoided. Indeed, side aggregation phenomena of graphene and reduced graphene flakes occur when concentrations higher than 0.16 mg/mL in water and 60 mg/mL in *N*-methylpyrrolidone (for graphene) are reached.^20,21^

**Figure 1:**
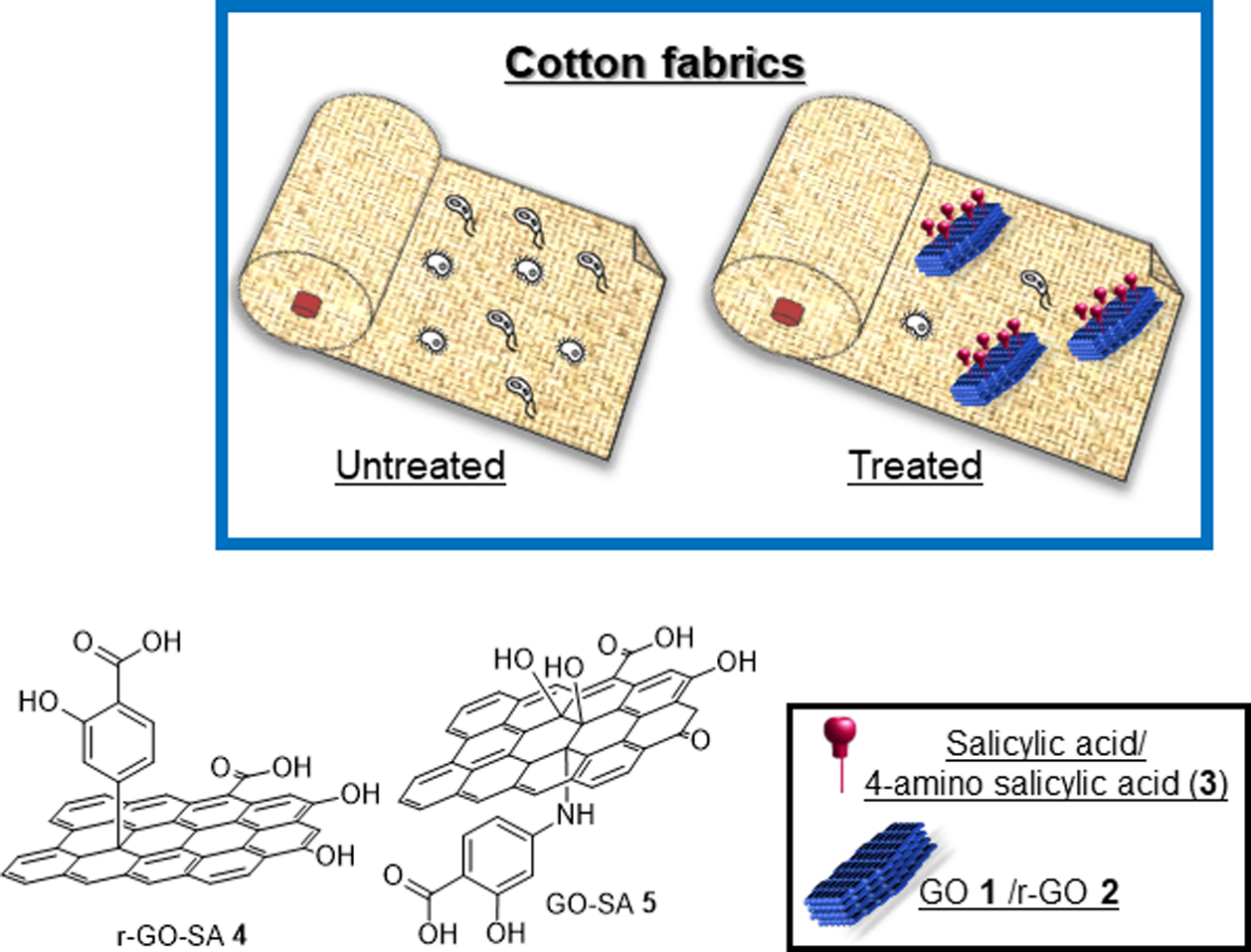
General structure of materials r-GO-SA **4** and GO-SA **5**. Schematic representation of cotton fabrics embedded with r-GO-SA **4** and GO-SA **5**.

In this framework, this work reports on the efficient production of graphene oxide (GO, **1**) and reduced graphene oxide (r-GO, **2**) functionalized with 4-aminosalicyclic acid (ASA) **3** (Figure 1). ASA-**3** has been chosen as it provides well-known intrinsic antimicrobial properties,^22^ and in addition it contains both hydroxyl and carboxylic groups, which can both promote graphene–bacteria physiochemical interactions and improve graphene dispersibility in water. Then, GO **1** and r-GO **2** are characterized by a different oxygen content and different hydrophilicity, thus allowing us to compare different functionalization approaches. Indeed, GO **1** was functionalized *via* a nucleophilic ring opening of the epoxide ring on the oxidized graphenic platform in a mechanochemical process, whereas r-GO **2**, due to its partially recovered extended p-system, was more conveniently functionalized *via* the classical Tour approach. Concerning the functionalization of r-GO or exfoliated graphene, this classical synthetic approach is alternative to the electrochemical exfoliation and functionalization using diazonium salts, which has also proved to be an efficient approach.^23,24^

In particular, we prepared the functionalized graphene-based materials r-GO-SA **4** and GO-SA **5** (Figure 1), by exploiting different surface chemistries (i.e., the conventional (in batch) solution-based Tour reaction and a mechanochemical approach) to produce up to 1.0 gram of functionalized materials. The functionalized materials have been embedded onto a sample of textile manufacture (i.e., cotton fabrics) and they proved to efficiently provide a significant antimicrobial activity avoiding variation of the textile properties. Crystalline nanocellulose (CNC)^25,26^ was used as an additive in the treatment of the fabrics. In this work, CNC plays two different roles (a) mimicking the cotton fabric in quartz crystal microbalance (QCM) experiments to assess the effect related to detergent treatments and (b) stabilizing the dispersion of the graphene materials and enhancing their interactions with the cotton fabric.

It is worth noting that a quartz crystal microbalance (QCM-D) on the CNC model system showed that the functionalization with the salicylic acid moiety significantly improved the physical interactions between graphene derivatives and cotton fibers even after surfactant solution treatments.

## Experimental Section

### Materials

Graphene oxide was purchased from NANESA as a spray dry powder GoNan (elemental analysis: C 38.92%; H 2.47%; N 0%) or as 0.4% w/w dispersion in water. Nanocrystalline cellulose (CNC) was purchased from Celluforce. All of the other reagents, for which synthesis is not described, were commercially available and had been used without any further purification.

### Sample Preparation

Transmission electron microscopy (TEM): the materials were dispersed in Milli-Q water at 0.2 mg/mL concentration and a volume of 5 μL was used for the analysis. UV–vis: the analysis was carried out on water dispersion at 0.05 mg/mL unless otherwise indicated.

### Synthesis of **2** (r-GO)^27^

A 1L round bottomed flask was loaded with 500 mg of **1** (spray dry powder), 2.0 g of sodium deoxycholate (SDC, Figure S17), and 500 mL of Milli-Q water (concentration: 1.0 mg/mL of **1**). The resulting mixture was dispersed using an ultrasonic bath (1 h, 59 Hz). Then, sodium carbonate (Na_2_CO_3_) was added until a pH of 8, followed by 1.28 mL of hydrazine solution (30% in water). The mixture was stirred at 80 °C for 12 h. During the time of the reaction, the color of the dispersion turned from brownish to black. The resulting dispersion was used for the preparation of r-GO-SA **4** (see below). An aliquot (10 mL) of the dispersion was dialyzed vs water and then lyophilized to afford a black powder, which was used for the characterization. UV–vis: λ_max_ = 271 nm (see the Supporting Information, SI Figure S1b). Fourier transform infrared spectroscopy (FT-IR): 3610, 1695, 1513, 1185, and 1079 cm^–1^ (Figure S1c). Elemental analysis: C 45.19%, H 0.77%, N 0.83%. TEM images are provided (Figure S2).

The same synthesis was performed without the use of SDC (using a more diluted dispersion of **1** (0.25 mg/mL) or substituting spray dry powder **1** with a cheaper (GO) water dispersion (0.4% w/w)) without appreciable differences (see the Supporting Information, page S3).

### Synthesis of **4** (r-GO-SA)^28^

Sodium nitrite (NaNO_2_, 231 mg, 0.98 mmol) and 4-aminosalicylic acid (150 mg, 0.98 mmol, **3**) were dissolved in 12.3 mL of a solution of NaOH (0.25% wt in water). The mixture was added dropwise to an ice-cooled HCl solution (0.1 M in water, 15.9 mL) under vigorous stirring. The resulting solution was added dropwise to a cooled dispersion of **2**-SDC (30 mg, 1 mg/mL) at pH 6 under vigorous stirring. The final dispersion was sonicated for 1 h (59 Hz) and then stirred at 80 °C for 12 h. The reaction mixture was filtered (0.4 μm polycarbonate membrane) and the filtrate was dispersed in water (1.0 mg/mL) and dialyzed (cellulose membrane cutoff = 14.000) vs water for 4 days to give 30 mg of pure r-GO-SA **4**. UV–vis: λ_max_ = 210 nm (Figure S5a). TEM images are provided (Figure S5b). Loading of salicylic acid based on thermogravimetric analysis (TGA) under a nitrogen atmosphere 4.70% w/w (Supporting Information, Figure S6).

The same procedure was also performed using r-GO **2** without the addition of SDC and r-GO **2** obtained from the reduction of a 0.4% w/w water dispersion of **1** (GO) without appreciable differences (see the Supporting Information, page S3).

### Mechanochemical Synthesis of GO-SA **5**

A 10 mL stainless-steel jar was filled with 200 mg of GO **1** (spray dry powder) and **3** in a 1:1 weight ratio. A steel ball of 1 cm diameter (26 g) was added and the jar was shaken for 40 min at 25 Hz. The jar was washed with Milli-Q water to recover the material and to obtain a 1.0 mg/mL dispersion of GO-SA **5**. The dispersion was dialyzed (14 kDa cutoff) until the UV–vis signal of **3** disappeared from the dialysis solution. The process was repeated four times to obtain four samples for which the elemental analysis is reported in Table S1. UV–vis: λ_max_ = 211, 261, and 299 nm (Figure S11a). FT-IR: 3222, 1735, 1616, 1232, 1074, and 781 cm^–1^ (Figure S11b), TGA (Figure S11c).

### Woven Fabrics Dyeing

Procedure: Samples of woven fabrics (7 × 3 cm) were immersed in the dispersion of selected nanomaterials and kept under stirring for 40 min. After that they were dried at room temperature for 30 min and washed with ethanol.

The following dispersions of selected nanomaterials were used:

Water dispersion of r-GO **2** and CNC: **2** 0.3 mg/mL, CNC 0.5% w/w.

Water dispersion of r-GO-SA **4** (with and without SDC) and CNC: r-GO-SA **4** 0.25 mg/mL, CNC 0.5% w/w.

Water dispersion of GO **1** and CNC: **1** 0.25 mg/mL, CNC 0.5% w/w.

Water dispersion of GO-SA **5** and CNC: **5** 0.25 mg/mL, CNC 0.5% w/w. On a visual analysis, due to the simple dyeing technique, none of the sample is completely homogeneous, although a higher homogeneity for fabrics dyed with functionalized GO and r-GO is evident (see images in Figure S13).

### Quartz Crystal Microbalance (QCM-D) and Film Preparation

QCM-D experiments were performed on a Q-Sense E1 instrument (Q-Sense, Gothenburg, Sweden) equipped with a 1 flow liquid cell (0.5 mL internal volume), containing a gold-coated quartz sensor with 4.95 MHz fundamental resonance frequency, mounted horizontally. Prior to use, the sensors were cleaned with ammonia/H_2_O_2_/water solution in a ratio of 1:1:5 for 5 min at 75 °C, washed with Milli-Q water and dried with nitrogen flux. After that a plasma cleaner was used for 10 min to completely oxidize the surface. Cellulose nanofilms on a sensor were prepared, as described by Gunnars and co-authors.^29^ First of all, an anchoring polymer (chitosan) was used to attach the cellulose onto the sensor, by dipping the sensor into a dilute solution of the polymer (0.01 g/L). After 15 min, the sensor was washed in deionized water at the same pH of the polymer solution and dried in an oven at 55 °C for 15 min. The cellulose’s film was produced using microcrystalline cellulose (CNC) dissolved in 50% wt *N*-methylmorpholine-oxide (NMMO) at 115 °C. Dimethyl sulfoxide (DMSO) was then used to decrease the viscosity. A thin layer of cellulose solution was spin coated at 2500 rpm with a hot solution of NMMO-CNC on a prechitosan-coated gold sensor. After extensively washing with water, the sensors were left to dry overnight in the fume hood.

The experiments were performed at 25 °C and the solvent exchange in the measurement chamber was achieved with a peristaltic pump. First, the sensor was placed in the chamber and water was injected at a low flow rate (0.07 mL/min), and the fundamental resonance frequencies (*f*) and corresponding energy dissipation factors (*D*) were measured for the odd overtones (1st–13th). A stable baseline for both *f* and *D* of the different harmonics was ensured before injection of the sample.

#### Detergent ECE B + Perborate

The detergent used contains a mixture of linear sodium alkylbenzenesulfonates and phosphates. Perborate was added as per standard procedure reports in a 1:4 ratio of ECE B. For the QCM tests, we prepared a solution of 2.6% w/w ECE B and 0.6% w/w perborate in Milli-Q water.

### Confocal Raman Microscopy

Raman spectra and Confocal Raman microscopy analysis were performed using an inVia Qontor confocal Raman microscope (Renishaw). The 532 nm laser line was used, in combination with an 1800 L/mm grating. The selected objective was ×50L (Leica). Spectra were acquired in the frequency range 102–3203 cm^–1^, with an exposure time of 10 s and 10% laser power. Confocal Raman mapping was performed in area scan mode, through a sequential acquisition of spectra from an array of sample points over a 15 μm × 15 μm area of the textile’s surface, with 2.5 μm spacing between the points. The collected spectra were analyzed to generate two-dimensional Raman images, with the color intensity at each pixel representing the integrated G-band intensity in the range 1453–1669 cm^–1^. Raman spectra and maps have been acquired directly on the cotton textile in the absence and in the presence of **1**, **2**, **4**, and **5**, without any other further treatment needed. Raman spectra of GO **1** and r-GO **2** powders are reported in Figure S14. Color maps of fabric + **1** and fabric + **2** are reported in Figure S15a,b, respectively.

### Bacterial Strains and Culture Conditions

The antimicrobial activity of the functionalized fabric pieces was determined against ATTC strains of *Klebsiella pneumoniae* (ATCC 700603), *Staphylococcus aureus* (ATTC 29213), and *Candida albicans* (ATTC 90028). All of the isolates were retrieved from frozen glycerol stocks, streaked on a fresh Mueller–Hinton (MH) agar plate, incubated at 37 °C for 18 h and subcultured to provide fresh colonies.

The different solid compounds, GO **1**, r-GO **2**, r-GO-SA **4**, and GO-SA **5** were dispersed in ultrapure distilled water to a final concentration of 256 μg/mL and sonicated in a water bath sonicator for 30 min before use. Broth microdilution susceptibility tests according to the European Committee on Antimicrobial Susceptibility Testing (EUCAST) international guidelines were performed. Tests were carried out on a 24-well plate by adding 200 μL of the microbial cell suspension equal to 5 × 10^5^ CFU/mL. Scalar dilutions between 128 and 2 μL/mL of each compound were added, and plates were incubated overnight at 37 °C. MCC values were determined by plating 5 μL of the content of each well on Mueller–Hilton agar plates that were incubated for 24 h at 37 °C. The MCC are defined as the lowest concentration corresponding to the death of 99.9% or more of the initial inoculum. Each test was performed in triplicate and both negative and positive controls were included.

### Biological Experiments with Fabrics

Fabric pieces of 1 × 1 cm were individually contaminated with 20 μL of the single microbe suspension containing 2.5 × 10^5^ colony-forming units (CFU)/mL of *K. pneumoniae*, *S. aureus*, and *C. albicans*.

The seeded gussets were incubated at 37 °C overnight and then transferred onto solid nutritive Mueller–Hinton (MH) agar by placing the contaminated side in contact with the solid nutrient medium for about 10 min. Then, each fabric was transferred into 500 μL of MH culture broth in a 24-multiwell plate and incubated, together with the MH Petri dishes, overnight at 37 °C. Each fabric piece was tested for sterility before the microbial contamination. Experiments were repeated in duplicate on different days.

### Scanning Electron Microscopy (SEM) Evaluation

All contaminated fabric pieces were investigated by SEM, (“Supra 24” Zeiss). Fixed and dried textiles were mounted onto an aluminum stub using double-sided carbon tape and coated with a gold/palladium film (80:20) using a high-resolution sputter coater (Agar Scientific B7234).

## Results and Discussion

### Synthesis of r-GO-SA **4** and GO-SA **5**

**Scheme 1:**
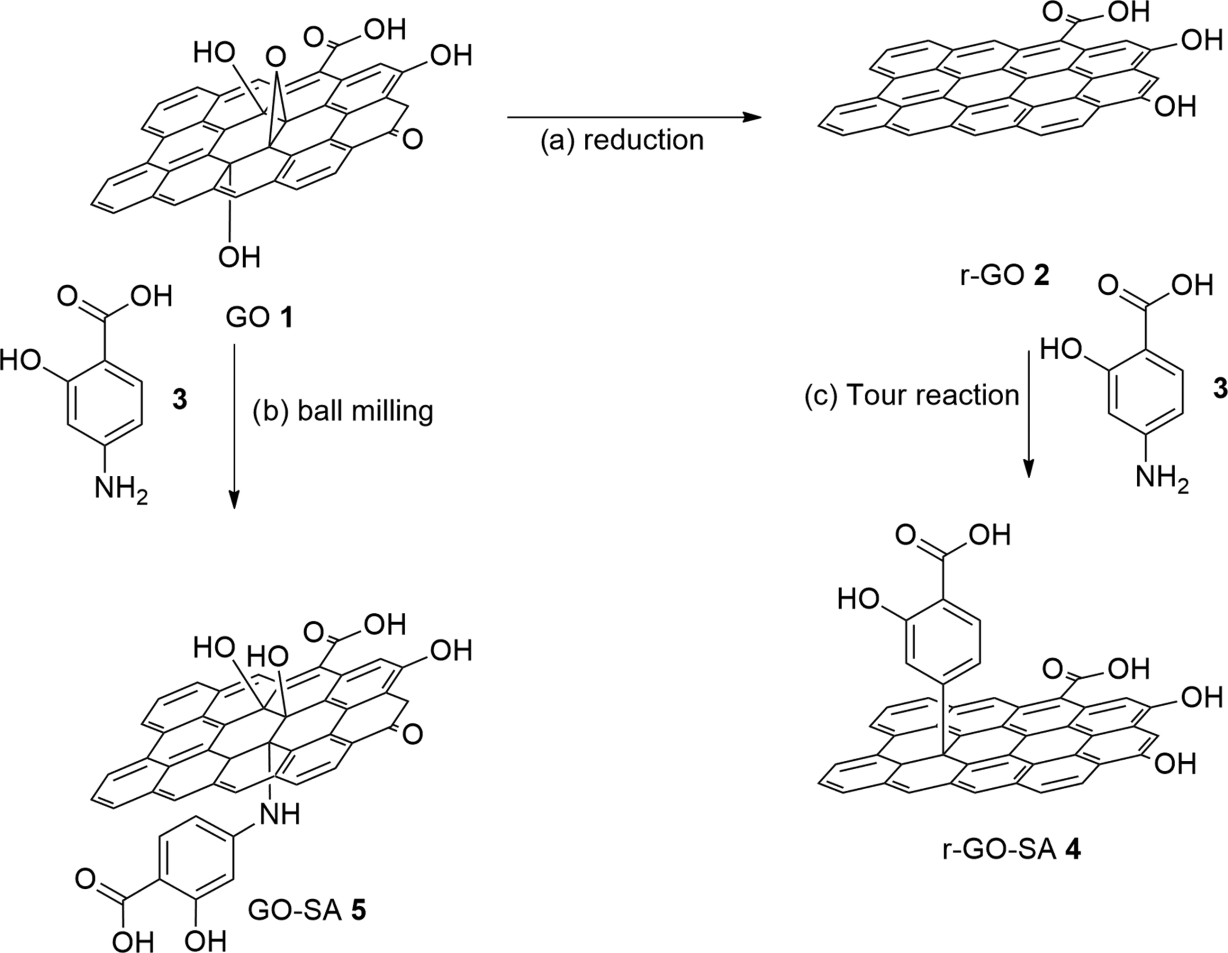
Modification of Graphene-Based Platforms (a) Graphene Oxide Reduction: See the Experimental Section; (b) **1**, 4-Aminosalicylic Acid (**3**) in a 1:1 Weight Ratio, 25 Hz, 40 min, and 25 mL Jars; and (c) Tour Reaction: See the Experimental Section

In Scheme 1, the synthetic strategy employed for the preparation of functionalized graphene-based r-GO-SA **4** and GO-SA **5** is described. These materials are structurally different in terms of the graphene platform employed and, accordingly, the type of covalent bond (C–C bond for r-GO-SA **4** and C–N bond for GO-SA **5**) between the graphene platform and the antibacterial agent. The synthetic process has been optimized in terms of reaction conditions (first experiments using isopentyl nitrite or *t*-butyl nitrite were unsuccessful, data not shown) and graphene concentration in the reaction mixture, paving the way for the scale-up of the process. Then, a significant degree of functionalization of the graphene platform has been reached by exploiting classical solution synthesis for r-GO **2** and a mechanochemical process for GO **1**.

The synthesis of **4** started with the reduction of **1** and the subsequent functionalization of the graphene reduced platform with salicylic acid moieties using 4-aminosalicyclic acid **3** in a Tour reaction. Similar approaches, using benzoic acid instead of salicylic acid residues, have been reported^28^ and they proceeded using exfoliated graphite flakes in *N*-methylpyrrolidone with *p*-amino benzoic acid.^28,30^ In these works, the highest concentration of graphene (alkaline pH due to the ammonia added to the reaction mixture) was achieved at 0.125 mg/mL, thus strongly limiting the scale-up of the process. Alternatively, the use of sodium dodecylbenzenesulfonate (SDBS) as the surfactant has been reported allowing us to reach higher concentrations (up to 0.4 mg/mL) of graphene during the functionalization step.^31^ However, the benzene ring of SDBS is itself prone to radical addition by aryl radicals produced during the reaction.^32^

In this work, the reduction of GO **1** with hydrazine has been accomplished modifying a reduction protocol reported in the literature,^33−35^ using sodium deoxycholate (SDC) instead of SDBS as the surfactant, to avoid radical side reactions. The reaction was monitored by UV–vis spectroscopy and, as expected, the UV maximum shifted from λ_max_ = 230 nm (λ_max_ conventionally related to GO)^30,36,37^ to a λ_max_ = 271 nm, which is usually associated with reduced graphene materials (Figure S1a). The selected surfactant stabilized the resulting r-GO **2** dispersion in water at a concentration up to 1.0 mg/mL. An analytical batch of **2** was purified by dialysis (10 kDa membrane cutoff) to remove SDC, lyophilized and then fully characterized (Figures S1 and S2). A transmission electron microscopy (TEM) analysis showed that **2** consisted mainly of monolayer flakes (Figure S2). The role of SDC was confirmed by the lower r-GO **2** concentration in the reaction medium (0.25 mg/mL) obtained in a separate experiment, which was performed without the use of SDC. In addition, the overlapping of the UV–vis spectra of the two batches of r-GO **2** (Figure S3 vs Figure S1) confirmed that SDC only affected the r-GO concentration in the reaction medium. The experiments were performed using the dry powder of GO but no apparent variation was observed using the cheaper and commercially available GO dispersion (0.4% w/w in water) (Figure S1 vs Figure S4). The IR spectra (Figures S1c, S3b, and S4b) showed the same main signals in the zone >3000 cm^–1^ due to the presence of hydroxyl groups left after the reduction and the main band near 1550 cm^–1^. r-GO **2** was the substrate for the Tour reaction that was performed to ensure a significant batch of functionalized nanomaterials in a congruent reaction volume. Specifically, the preparation of the diazonium salt was carried out modifying the protocol reported by Wei et al.^28^ Due to the low stability of the dispersion of **2** in acidic medium, the diazonium salt of **3** (5-fold excess in weight compared to **2**) was prepared in a separate flask and slowly added using a cannula in a cooled aqueous dispersion of **2** and SDC (see the Experimental Section). The material was filtered over a hydrophilic polycarbonate membrane (0.4 μm) and the solid was washed thoroughly with Milli-Q water until a colorless solution was obtained. The use of SDC combined with a dispersion of **2** (pH 6) allowed the production of up to 500 mg of functionalized r-GO-SA **4** (Scheme 1) by maintaining a congruent reaction volume (1.0 mg/mL, see the Experimental Section). Then, the functionalized r-GO-SA **4** was fully characterized by means of UV–vis, FT-IR spectroscopy (Figures S5–S8), and transmission electron microscopy (TEM) analysis (Figure S5b). The UV spectrum (Figure S7) showed a new band (211 nm) due to the salicylic acid moiety linked to the graphenic platform while the IR spectrum (Figure S8b) did not show relevant differences resected from the starting material r-GO 2 (Figure S1c). The thermogravimetric analysis (Figure S6a) confirmed a good loading of salicylic acid (0.34 mmol/mg, 4.70% w/w). It should be noted that to ensure no residual SDC affected the thermogravimetric outcome, a control experiment, without SDC, was performed, and TGA analyses of the two adducts were compared (Figure S6).

Both short- and long-term colloidal stability of **4** (1.0 mg/mL in water) was assessed by closely monitoring the absorbance and hydrodynamic size over time (Figures S9 and S10). Data obtained showed that the UV absorbance slowly decreased over the first 10 days by reaching a 0.5 mg/mL concentration that remained stable for over 2 months (Figures S9 and S10).

The functionalized GO**-**SA **5** was obtained starting from GO powder and 4-aminosalicylic acid **3** by means of a green and solvent-free mechanochemical process, exploiting the nucleophilic substitution of the amino group of **3** on the epoxide groups on the GO platform. Specifically, the synthesis of **5** was based on a dry ball milling reaction of **1** and **3** in a 1:1 weight ratio and grinding in a 10 mL stainless-steel mixing mill with a 1 cm Ø stainless-steel sphere for 40 min at 25 Hz. The black powder was easily recovered from the jar, and it was dispersed in Milli-Q water (1.0 mg/mL) and dialyzed (14 kDa cutoff) until no UV–vis signal of **3** was recovered in the dialysis solution. Then, **5** was fully characterized by means of UV–vis and FT-IR spectroscopy (Figure S11). Notably, both thermogravimetric analysis (Figure S11) and elementary analysis (Table S1) confirmed a significant loading of salicylic acid, which shifted from 0.34 mmol/g of the Tour process up to 1.47 mmol/g in the ball milling process (4.70 vs 22.6% in weight).

### Quartz Crystal Microbalance (QCM) Measurements: Adsorption of Graphene-Based Materials **4** and **5** onto a Model System of Cotton Fabric

Despite the numerous studies that make use of graphene and graphene oxide adsorbed onto textiles, a quantitative mechanistic study addressing the adsorption process of GO **1** and r-GO **2** onto model textiles is still missing. We used a quartz crystal microbalance (QCM) to explore the adsorption of GO **1**, r-GO **2**, and their derivatives **4** and **5** onto crystalline nanocellulose (CNC) fibers, used as a model of cotton fibers, deposited onto a gold-coated QCM sensor.

**Figure 2:**
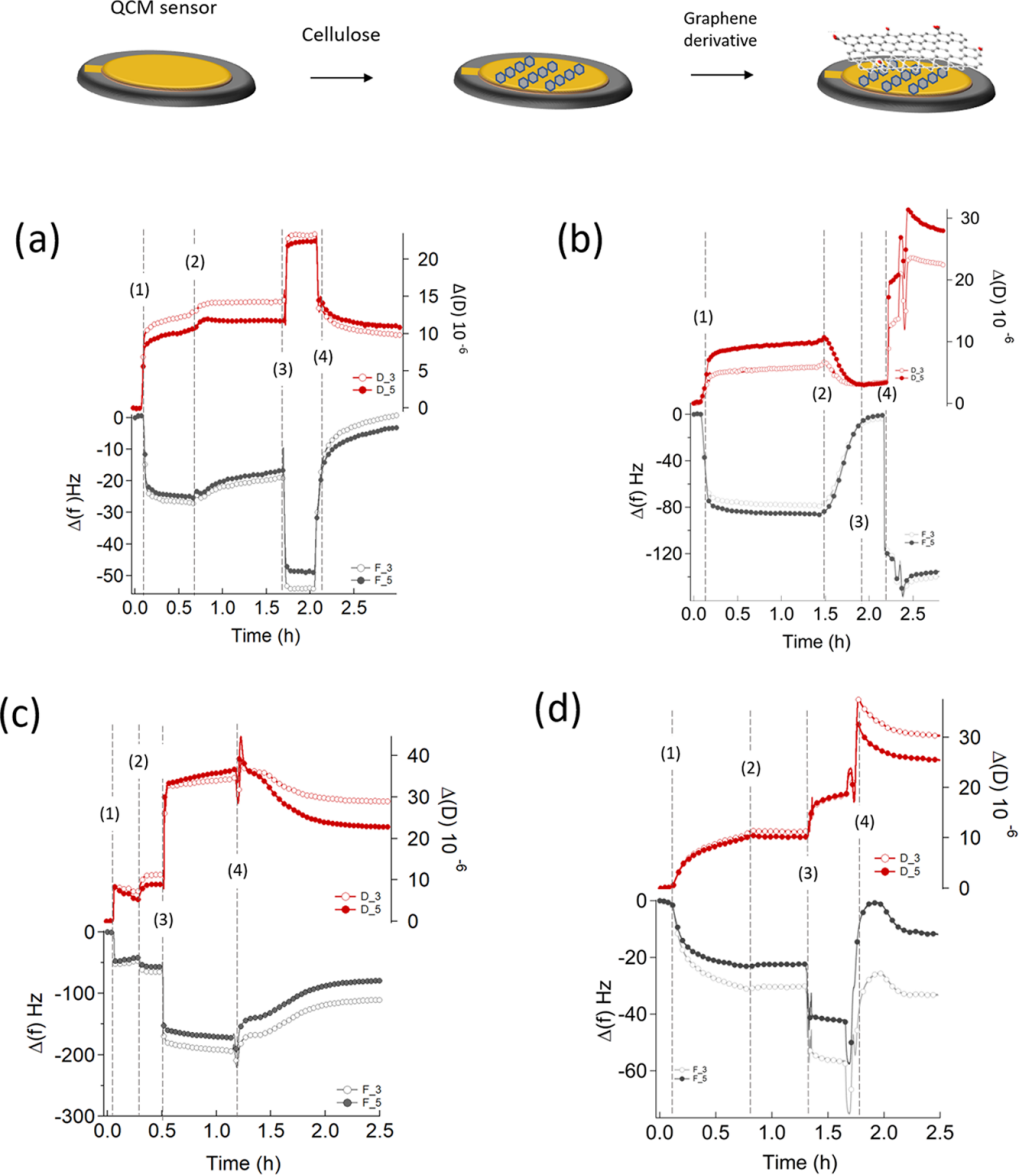
QCM-D frequency shift (Δ*f*) and dissipation (Δ*D*) for the third and fifth overtones as a function of time during the formation of the adlayer (a) GO **1**, (b) r-GO **2**, (c) GO-SA **5**, and (d) r-GO-SA **4**. Injection of samples (step 1) was followed by Milli-Q water rinsing (step 2). Then, an ECE B and sodium perborate-based detergent was flushed into the measuring chamber (step 3), followed by a second rinsing step with Milli-Q water (step 4).

The sensors, cleaned and coated with a chitosan film, were put in contact with a solution of CNC dissolved in 50% wt *N*-methylmorpholine-oxide (NMMO) at 115 °C. After extensive water rinsing, the sensor was left to dry overnight in the fume hood. The sensor was then placed in the measurement chamber and water was added at a low flow rate (0.07 mL/min); the fundamental resonance frequencies (*f*) and the corresponding energy dissipation factors (*D*) were measured for the odd overtones (1st–13th). A stable baseline for both *f* and *D* of the different harmonics was ensured before injection of the sample. Then, 500 μL of a water dispersion of different samples (i.e., **1**, **2**, **4**, and **5**) at a concentration of 0.05 mg/mL was injected in the measuring chamber (step 1, Figure 2); the adsorption of the graphene derivatives was monitored by recording the variations in *f* (Δ*f*) and *D* (Δ*D*) of the different harmonics. To check the stability of adsorption, the samples’ injection and equilibration were followed by: (i) a first rinse with Milli-Q water (step 2, Figure 2); (ii) a subsequent injection of a detergent (step 3, Figure 2), commonly used as a standard for color fastness to domestic and commercial laundering, containing 2.6% wt ECE(B) (a phosphate-based detergent powder) and 0.6% wt sodium perborate; and (iii) a final rinse with Milli-Q water (step 4, Figure 2), which removes the detergent and possibly the adsorbed layer (adlayer) of graphene derivatives from the substrate.

In a QCM-D experiment, Δ*f* is related to the mass variation^38−40^ while Δ*D* can be used for a qualitative profiling of structural changes in the system, in terms of viscoelastic properties of the film. A decrease in Δ*f* implies mass addition to the sensor, whereas an increase in Δ*D* indicates that the system has become less compact.^40^

For every graphene derivative, the sample injection always leads to an increase (in absolute values both of the frequency and of the dissipation factor, indicating adsorption on the CNC substrate (step 1, Figure 2)). For r-GO **2** dispersion, an almost complete desorption takes place after the first rinse with Milli-Q water (step 2, Figure 2b), with frequency and dissipation recovering the initial values of the CNC substrate. On the other hand, for GO **1** dispersions, a practically complete desorption occurs after injection of the detergent solution (step 3, Figure 2) and subsequent rinsing with Milli-Q water (step 4, Figure 2), pointing out a stronger interaction of this derivative with the CNC layer, which we attribute to the higher hydrophilicity of graphene oxide with respect to the reduced form that increases the affinity for cellulose (Figure 2a). Conversely, the adsorption of the graphene derivatives decorated with salicylic residues (**4** and **5**) results in the formation of a homogeneous layer across the support, which is conserved after injection of the detergent and rinsing with Milli-Q water (Figure 2c,d, step 3 and 4).

**Figure 3:**
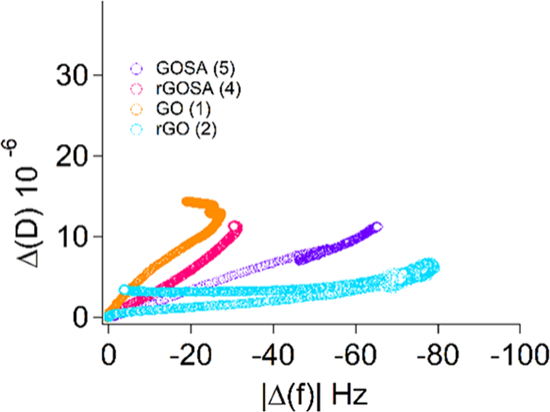
QCM-D dissipation (Δ*D*) vs frequency shift (Δ*f*) for the third overtone for all measured samples after the first rinse with Milli-Q water, before injection of the detergent solution. It is evident from the curves that the most rigid layer is formed in the case of r-GO **2** dispersion.

Concerning the variation of the dissipation factor, the adsorption of GO **1** (Figure 2a) leads to a nonrigid layer, as evident from the increase in dissipation as well as from a non-negligible difference between the overtones.^40^ The soft nature of the layer is not modified by Milli-Q water rinsing (step 2, Figure 2). On the contrary, r-GO **2** (Figure 2b) adsorption leads to a soft layer, which becomes more rigid, i.e., more compact after water rinsing (Figure 2b). We ascribe these differences in rigidity as due to a different hydration of the adlayer. For SA-decorated graphene derivatives **4** and **5** the behavior is similar, with the difference between overtones after water rinsing more pronounced for GO-SA **5** with respect to r-GO-SA **4** (Figure 2c,d), confirming the hypothesis mentioned above. Figure 3 reports the dissipation factor as a function of the frequency taken from the beginning of the measurement until the end of the second step (i.e., first rinsing with Milli-Q water): in line with the above observations, r-GO **2** forms a more rigid layer and exhibits a lower adsorbed mass, while GO **1** is associated with a softer layer, with a higher adsorbed mass. Also in this case, this behavior also holds in the case of functionalization with salicylic residues.

The injection of the detergent solution, performed to evaluate the resistance of the adsorbed layer on CNCs, leads to the formation of a stable, nonrigid layer in all of the systems (step 3, Figure 2) in all of the panels of Figure 2. After rinsing with Milli-Q water (step 4, Figure 2), the GO **1-**coated sensor exhibits a decrease in the absolute frequency (step 4, Figure 2a) highlighting a loss of both the surfactant and GO **1**, reaching the quasi-complete removal of the GO layer after 1 h of washing time.

On the contrary and notably, the presence of SA on both GO and r-GO samples **4** and **5** improves the resistance of the adsorbed graphene layer on CNCs, which appears higher for GO-SA **5** (Figure 2c) with respect to r-GO-SA **4** (Figure 2d): for a long rinsing time, we observe a stable Δ*f* of roughly −130 ± 4 and −40 ± 3 Hz for GO and r-GO, respectively. Furthermore, for the case of r-GO-SA **4** (Figure 2d), the second rinse leads to detergent removal, while in Figure 2c we observe a higher affinity of the detergent with the GO-SA **5** adlayer, probably due to the formation of Van den Waals interaction between the adsorbed molecules.

QCM-D experiments can also provide qualitative information on the depth profiling through a comparison of the frequency changes of the different harmonics.^41−44^ Each harmonic probes a defined distance away from the surface of the sensor, inversely proportional to its frequency^45^ so that higher harmonics probe a closer distance to the sensor surface. Thus, this comparison provides an assessment of the nature of the interaction of the graphene derivatives with the cellulose layer, i.e., surface vs translayer binding.

In all of the measurements, we notice that the presence of salicylic residues restricts the interaction of graphene derivatives closer to the surface (Figure S12), while for GO **1** the higher harmonics are more sensible to the mass loading confirming that there is a translayer binding. Due to the poor adsorption of r-GO **2** on the surface, as mentioned above, no conclusions can be gathered in terms of depth profiling.

### Dyeing of Cotton Fabrics

Cotton is the most widely used natural fiber to produce fabrics that possess comfort, breathability, and low price. However, cotton textiles can easily store humidity and become a growth medium for microorganisms. For these reasons, significant efforts have been devoted to developing cotton treatments to confer antimicrobial properties to the fibers.^46^ The ideal antimicrobial cotton fibers should contain washing-resistant additives that do not alter the product properties and are active at low concentrations to minimize side effects. In this work, a cotton woven fabric (100% cotton) was used, and the ability of functionalized graphene materials **4** and **5** to be adsorbed onto the fabrics and thus, to provide antibacterial activity was evaluated. These fabrics (216 g/m^2^) are characterized by a plain weave.

Based on the results obtained from the model systems of cotton fibers, we used a simple protocol based on physical adsorption of a CNC dispersion (0.5% w/v in water) of the graphene material **4** or **5** (0.25 mg/mL) onto the cotton fabrics. CNC was used to optimize the adsorption of the materials, **1, 2, 4**, and **5**, onto the fabrics. Thus, a fabric sample (7 × 3 cm) was immersed in a graphene dispersion (120 mL, 0.25 mg/mL) and left under stirring for 40 min. Then, cotton fabrics were dried at room temperature for 30 min, copiously rinsed with absolute ethanol and dried again at room temperature (Figure S13). Then, fabrics embedded with the antimicrobial fillers were characterized by means of confocal Raman microscopy. Raman spectra have been acquired directly on the textile, before and after the treatment with GO **1**, r-GO **2**, r-GO-SA **4**, and GO-SA **5**, to verify the absorption of samples and explore their distribution within the fabric.

The typical Raman spectrum of graphene-based materials contains three main diagnostic bands, marked as D, G, and 2D.^47^ The D band (located near 1350 cm^–1^) results from the presence of vacancies and defects in the material. The next band, the G peak, is related to the in-plane vibration of sp^2^ hybridized carbon atoms and is located near 1580 cm^–1^. The last peak (2D) is related to the number of graphene layers and is located near 2700 cm^–1^.

**Figure 4:**
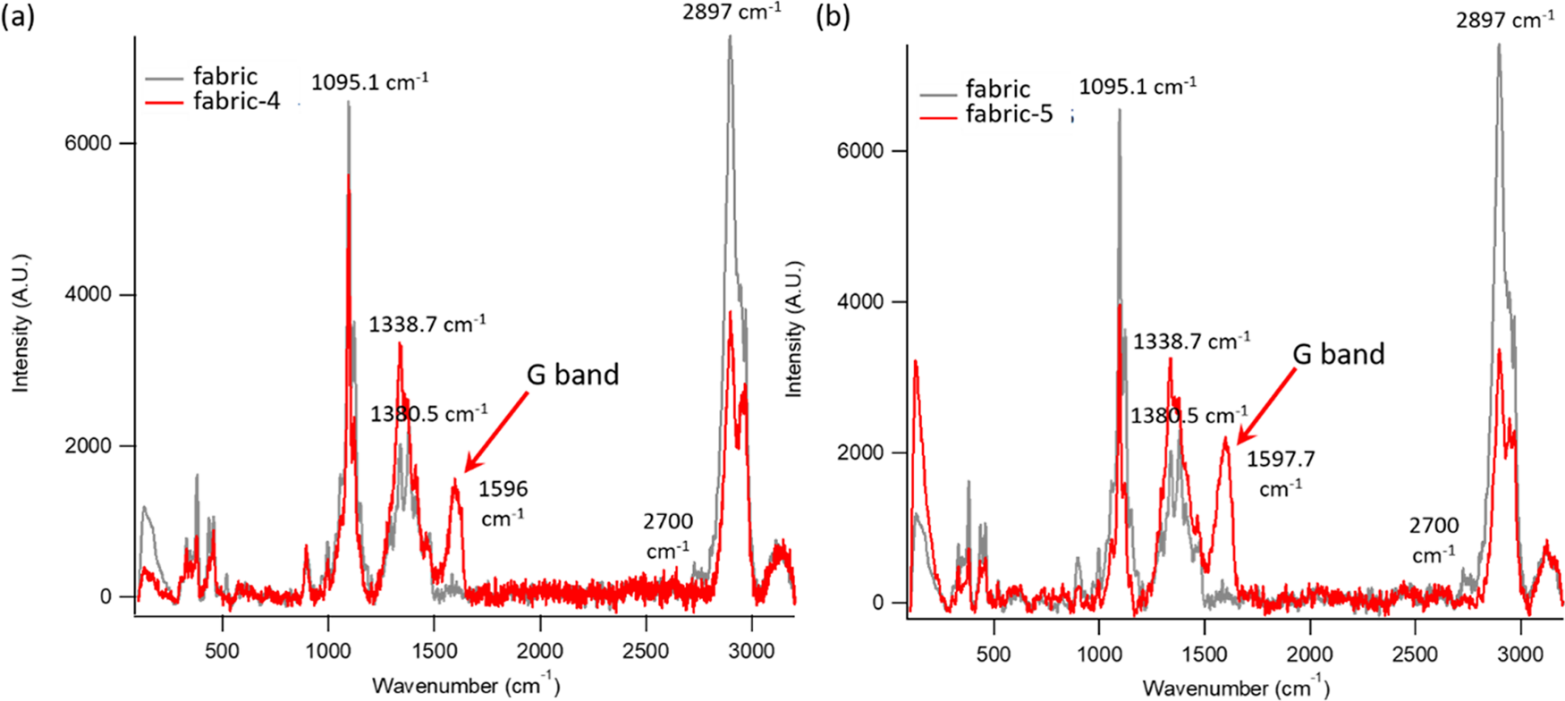
Raman spectra of (a) fabric and fabric + **4** and (b) fabric and fabric + **5**.

Figure 4 shows the Raman spectra registered on the original cotton fabric and on the same fabric after dyeing with r-GO-SA **4** (Figure 4a) and GO-SA **5** (Figure 4b), for the laser excitation wavelength λ_max_ = 532 nm. In the frequency range 102–3203 cm^–1^, the starting sample of the fabric showed several bands, the most intense of which were located at 1095, 1380, and 2897 cm^–1^ (gray spectrum in Figure 4a,b). The decoration of the fabric with both r-GO-SA and GO-SA leads to the insurgence of an additional intense Raman signal, located at 1594 cm^–1^, absent for the original fabric; this signal, also present in the Raman spectra of pure GO and r-GO powders (Figure S14), can be unambiguously assigned to the G band of graphene, proving the effective adsorption of the graphene derivatives on the fabric. The D band of graphene, clearly detectable in the spectra of pure GO and r-GO, cannot be easily identified in Figure 4a,b, due to the overlap with the wide band centered at 1380 cm^–1^, characteristic of the fabric. The 1597 cm^–1^ Raman band, characterized by high intensity and unambiguously ascribed to the nanostructured material, can act as a selective probe for mapping the distribution of the graphene derivatives with and without salicylic residues on the fabric. To this end, confocal Raman microscopy was used in the area scan mode, to locate graphene derivatives over the fabric surface, after treatment with **4** and **5**. Raman mappings were performed over the fabric’s surface, using a λ_max_ = 532 nm incident wavelength. In the mapping, Raman spectra were sequentially acquired from an array of sample points spanning a 15 μm × 15 μm area of the textile’s surface, with 2.5 μm spacing between points (see the SI for further details). The collected spectra were analyzed to generate two-dimensional Raman images, where the color intensity at each pixel details the integrated G-band intensity in the range 1453–1669 cm^–1^.

**Figure 5:**
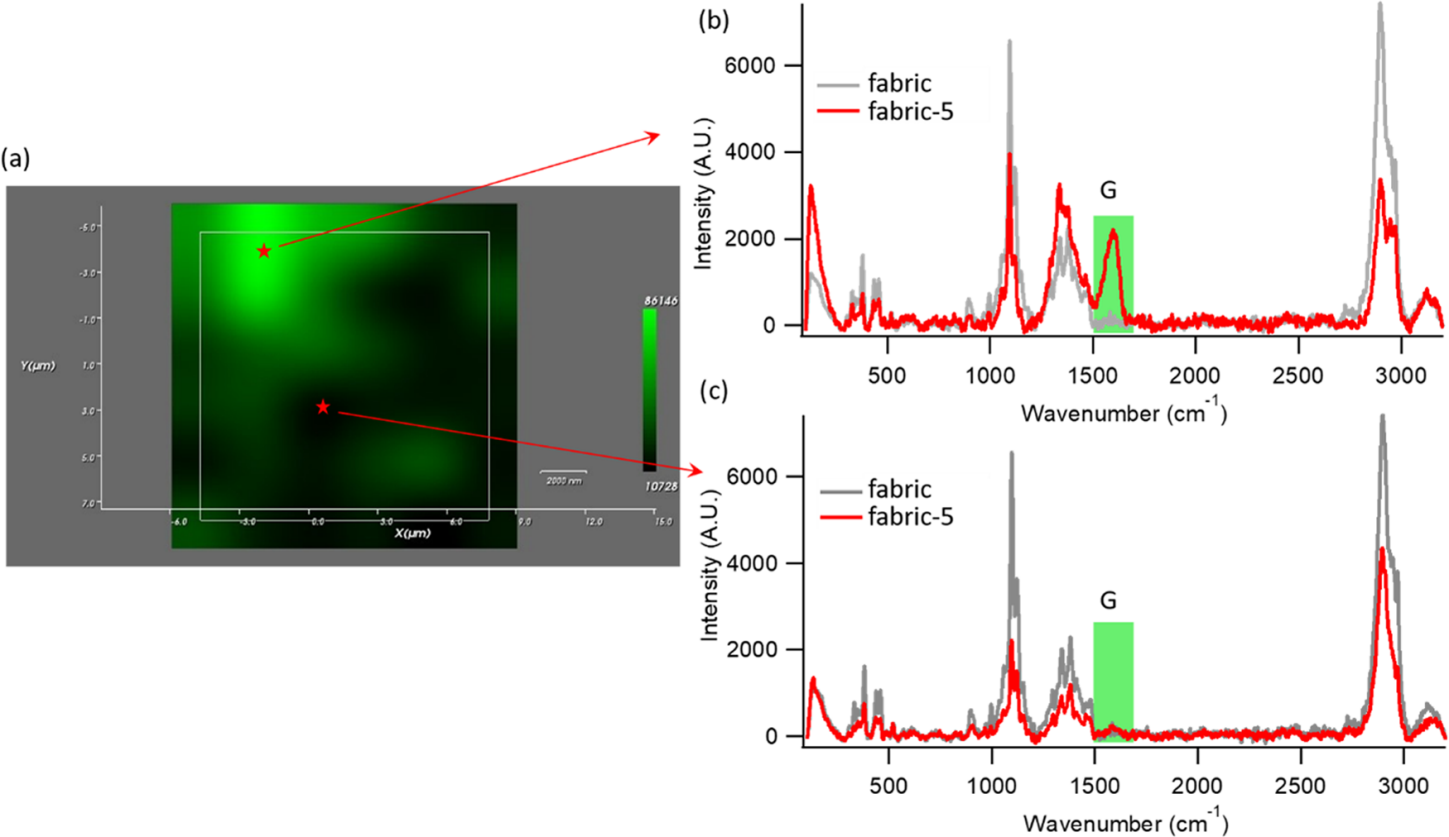
(a) Raman map of the integrated G line intensity of graphene (1453–1669 cm^–1^) over the surface of the fabric, treated with **5**. (b and c) Representative Raman spectra from which the color map of fabric + **5** is obtained, corresponding to (b) a high color intensity point in the Raman map (the high intensity arises from the intense G band, highlighted in green in the spectrum) and (c) a black region of the Raman map (associated with the nondetectable G-band-related signal in the Raman spectrum).

Figure 5a represents the color maps obtained for Rublo dyed with **5** and shows the distribution of the graphene derivatives on the textile. The variations in the color intensity reflect different intensities of the G band recorded throughout the mapped area, with bright green regions in the map color scale corresponding to higher intensities (Figure 5b) and black points indicating the nondetectable signal in the same spectral range (Figure 5c). The map image clearly shows the successful decoration of the fabric with GO-SA **5** covering extensive portions of the fabrics. The color map obtained for the fabric dyed with **4** (see Figure S16 of the SI) reports a very similar behavior, with r-GO-SA extensively covering the textile surface. Color maps of fabrics dyed with **1** and **2** were also acquired as control samples (Figures S14 and S15), showing a similar distribution of samples over the fabric surface.

### Evaluation of Antimicrobial Activity of the Treated Cotton Fabrics

Minimal Cytocidal Concentration of functionalized (**4** and **5**) and unfunctionalized graphene materials (**1** and **2**) were evaluated against strains of *K. pneumoniae andS. aureus* as representatives of, respectively, Gram negative (Gram (−)) and Gram positive (Gram (+)) bacteria strains and *C. albicans* as representative of a fungus. Data obtained (Table S2) confirmed that all of the materials were active at a 128–2.0 μg/mL concentration range. Particularly, r-GO-SA **4** showed the highest antimicrobial activity when compared with other materials (Table S2). On the basis of these data, graphene-embedded fabrics (Table 1) were then tested (see the SI for details) for their antibacterial activity against the same strains.

**Table 1:** Antibacterial Activity of Graphene-Embedded Rublo Fabrics against Strains of Bacteria (Gram + and Gram −) and Fungus

entry	additives	*K. pneumoniae*	*S. aureus*	*C. albicans*	CTR–
1	CNC	++++	++++	+++	–
2	SDC	++++	+++	+++	–
3	r-GO **2** (with SDC)	–	++	–	–
4	r-GO-SA **4** (no SDC)	++++	++++	+	–
5	r-GO-SA **4** (with SDC)	–	+	+	–
6	GO **1**	–	++	+	–
7	GO-SA **5**	–	+++	+	–

a Notes: (−) no growth, (+) from 3 to 10 colonies, (++) weak growth, (+++) moderate growth, and (++++) spreaded growth.

Data showed that the fabric treated with a CNC (0.5% in H_2_O) dispersion had no inhibitory effect on microbial growth (entry 1, Table 1). The same result occurred with fabrics treated with a SDC solution (4.0 mg/mL in H_2_O) (entry 2, Table 1). However, the critical role of SDC became evident by comparing entry 4 with entry 5 (Table 1). Indeed, fabrics treated with the r-GO-SA **4** dispersion in the absence of the surfactant (SDC), showed no activity (entry 4) toward either Gram (+) or Gram (−) bacteria. Conversely, the presence of SDC in the dispersion of r-GO-SA **4** (entry 5) ensured a protective effect against *K. pneumoniae* and only a few colonies of *S. aureus* have been observed as the readout of a significant antimicrobial activity.

On the other hand, both treatments proved effective in the inhibition of the growth of *C. albicans* (entries 4–5, Table 1). It should be noted that the presence of salicylic acid residues on graphene surface resulted in a slightly increased antimicrobial activity toward *S. aureus* (entry 5 vs entry 3, Table 1). Entries 6 and 7 (Table 1) show the results obtained with fabrics treated with, respectively, the GO **1** and GO-SA **5** dispersions. As expected, GO **1** induced a significant inhibition of microbial growth (entry 6, Table 1), hence the presence of salicylic acid in GO-SA **5** (entry 7, Table 1) adds a minor contribution.

In conclusion, both *S. aureus* and *C. albicans* showed a moderate growth on functionalized fabrics. Cell walls of the three microorganisms assayed have a highly peculiar structure and composition and probably these differences impact on the different degree of inhibition observed. *C. albicans* has a complex cell wall consisting of an outer layer of mannans and an inner layer of β-glucans and chitin while the staphylococcal cell wall is composed of a thick and highly cross-linked A3α-type peptidoglycan. The cell wall of Gram- *K. pneumoniae* is composed of a thin, inner layer of peptidoglycan and an outer membrane consisting of molecules of phospholipids enriched with lipopolysaccharides. GO and r-GO are known to interact with the microbial surface causing mechanical breakdown and leakage of the cell content.^48^ In addition antimicrobial properties of SA make the mechanism of the action of this nanocomposite even more complex.

**Figure 6:**
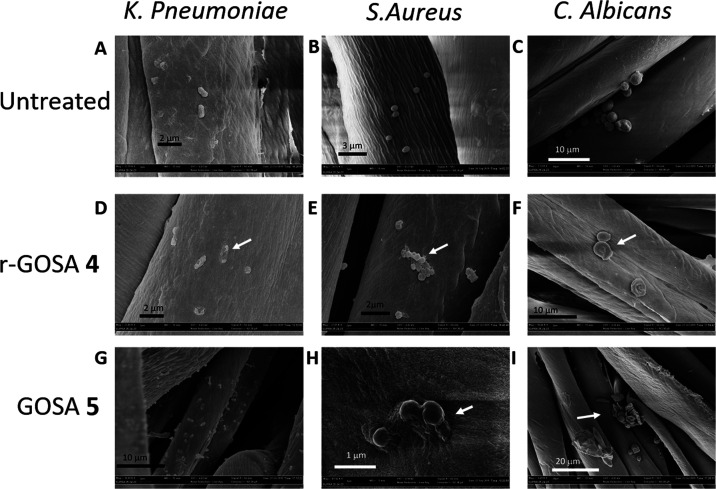
SEM representative images with different magnifications of bacterial cells after growth on the control and functionalized cotton fabrics. (A, B, and C) *K. pneumoniae*, *S. aureus,* and *C. albicans,* respectively, on fabrics (negative control); (D, E, and F) *K. pneumoniae*, *S. aureus,* and *C. albicans,* respectively, on r-GO-SA **4** embedded fabrics; and (G, H, and I) *K. pneumoniae*, *S. aureus*, and *C. albicans,* respectively, on the GO-SA **5** embedded Rublo fabric. White arrows indicate damaged microbial cells.

To evaluate the alteration of the cell morphology of the three microorganisms (i.e., *K. pneumoniae*, *S. aureus,* and *C. albicans*) scanning electron microscopy (SEM) images of the graphene-embedded fabrics were recorded (Figure 6).

Figure 6A–C show the cell morphology of *C albicans*, *S. aureus,* and *K. pneumoniae* on fabrics. As expected, no evident alterations were observed for all of the three microorganisms studied, as proof of the absence of cellular suffering. Conversely and according to results obtained by in vitro antimicrobial analyses, all microorganisms showed an altered morphology when grown on fabrics treated with either r-GO-SA **4** or GO-SA **5** (Figure 6D–I). Specifically, the morphology of *C. albicans* cells (Figure 6F,I) is significantly altered, indeed, several yeasts are deflated and have no peculiar rounded structure. Then, ghost yeast cells, which have completely released the cellular content, are frequently present. Some other cells have a partially modified morphology even if in a less evident way. r-GO-SA **4** and GO-SA **5** also interfere with the growth of *S. aureus* on fabrics (Figure 6E,H) and, despite some staphylococci maintaining a rounded cellular shape, morphological features of several bacterial cells appeared as deflated and lysed indicating clear cell suffering. Then, the Gram (−) bacterium *K. pneumoniae* appears clearly altered on fabrics treated with r-GO-SA **4** and GO-SA **5** (Figure 6D,G) with a morphology closely related to dead cells and with a partial preservation of the elongated shape of the cell body. Then, notably, the average cell number on decorated fabrics is lower than that shown for the control fabrics. The SEM images in Figure 6 show convincing evidence that the microbial morphology is altered.

## Conclusions

The functionalization of reduced graphene oxide and graphene oxide is a useful approach for the preparation of metal-free antibacterial additives that can be used directly on cotton fabrics. Reliability, eco-compatibility, and scaling-up of the process are key points which once addressed can pave the way for the translation of the use of functionalized graphene materials from the bench to industrial-driven application. In this context, we demonstrated that the functionalization of structurally different graphene materials can be achieved by exploiting a gram-scale synthetic process which uses, accordingly with the protocol employed, or water as the solvent in a reduced and optimized volume scale, or mechanochemical forces in an eco-friendly process. Despite the presence of salicylic acid moieties on the graphene platform slightly improving the antibacterial activity, it surprisingly increased the interactions of the graphene materials with the cotton fabric as demonstrated by a quartz microbalance study. Graphene versatility can be exploited for the development of new nanoengineered antibacterial cotton materials for a wide range of applications, including antimicrobial gowns in healthcare settings.

## Supporting Information Available

Chemicals and materials; characterization and experimental methods; and TEM, UV–vis spectra, Raman spectra, and TGA analyses (PDF)
