# Supplementary material for: Metal-Free Antibacterial Additives Based on Graphene Materials and Salicylic Acid: From the Bench to Fabric Applications
Source: ACS Appl Mater Interfaces. 2021 May 26;13(22):26288–98. doi: 10.1021/acsami.1c02330 (PMC8289172; doi:10.1021/acsami.1c02330)
Supplement: Supplementary file 1 — am1c02330_si_001.pdf [file am1c02330_si_001.pdf]

Supporting information

Metal-free antibacterial additives based on graphene materials and salicylic acid: from the bench to fabrics applications.

Giacomo Biagiotti,[a,b] Annalisa Salvatore,[c,£] Gianluca Toniolo,[a,b] Lucrezia Caselli,[a,c] Maura Di Vito,[d,e] Margherita Cacaci,[d,i] Luca Contiero,[f] Tommaso Gori,[g] Michele Maggini,[h] Maurizio Sanguinetti,[d,i] Debora Berti,[a,c] Francesca Bugli,[d,i] Barbara Richichi,[a,b,,#] Stefano Cicchi.[a,b,*#]*

a) Department of Chemistry, Università di Firenze, Via della Lastruccia 3-13, 50019, Sesto Fiorentino, Italy ; b) INSTM (Consorzio Interuniversitario Nazionale per la Scienza e Tecnologia dei Materiali) Via G. Giusti, 9, 50121 Firenze (ITALY); c) CSGI (Italian Center for Colloid and Surface Science, Via della Lastruccia 3, Sesto Fiorentino, 50019 Firenze, Italy; d) Dipartimento di Scienze Biotecnologiche di Base, Cliniche Intensivologiche e Perioperatorie, Università Cattolica del Sacro Cuore, Rome, Italy; e) Dipartimento di Scienze e Tecnologie Agro-Alimentari, Università di Bologna, Viale G. Fanin 42, 40127 Bologna, Italy; f) Cromology Italia S.p.A., Via IV Novembre, 4, 55016 Z.I. Porcari (Lucca); g) Beste S,p,A Via Primo Levi, 6 59022 Colle Cantagallo - Prato – Italy; h) Dipartimento di

Scienze Chimiche, Università degli Studi di Padova, Via Marzolo 1, 35131, Padova, Italy; i)

Dipartimento di Scienze di Laboratorio e Infettivologiche, Fondazione Policlinico

Universitario A. Gemelli IRCCS, Rome, Italy.

£ Present address: Evotec, 111 Innovation Drive, Milton Park, Abingdon, Oxfordshire OX144RZ, England, UK

#B.R. and S.C. are co-last and co-corresponding authors

Corresponding authors: stefano.cicchi@unifi.it; barbara.richichi@unifi.it.

Contents

Figure S1. UV-Vis spectrum of a) GO (0.05 mg/mL in H_2O); b) r-GO **2** (0.05 mg/mL in H_2O) prepared from GO powder (λ_{max} = 271 nm) with SDC. c) FT-IR spectrum of r-GO **2,** KBr tablet prepared from GO powder. S-4

Figure S2. TEM image of r-GO **2** prepared from GO powder. S-4

Figure S3. A) UV-Vis spectrum of rGO **2** (0.05 mg/mL in H_2O) prepared from GO powder without SDC (λ_{max} = 273 nm). B) FT-IR spectrum of r-GO **2** KBr tablet prepared from GO powder without SDC. S-5

Figure S4. A) UV-Vis spectrum of r-GO **2** (0.05mg/mL in H_2O) prepared from GO solution (0.4 % wt water GO). (λ_{max} = 269 nm). B) FT-IR spectrum of r-GO **2** KBr tablet prepared from GO solution (0.4 % wt water GO). S-5

Figure S5. A) UV-Vis spectrum of r-GO-SA **4** (0.05mg/mL in H_2O); λ_{max} = 269, 211 nm b) TEM image of r-GO-SA **4**. S-5

Figure S6. TGA analysis under nitrogen atmosphere of r-GO-SA **4**: a) with SDC (r-GO-SA **4 a**) (0.34 mmol/g, 4.70 % w/w) and b) without SDC (r-GO-SA **4 b**) (0.39 mmol/g, 5.39 % w/w). The weight loss calculated between 150 and 600 °C. S-6

Figure S7. UV-Vis spectrum of r-GO-SA **4** S-6

Figure S8. a) UV-Vis spectrum of r-GO-SA **4** (0.05mg/mL in H_2O); λ_{max} = 269, 210 nm. b) FT-IR spectrum of r-GO-SA **4** KBr tablet. S-7

Figure S9. a) Intensity of UV-Vis spectrum of r-GO-SA **4** dispersion (0.05 mg/mL) measured at 270 nm over time. S-7

Figure S10. Hydrodynamic size of r-GO-SA **4** dispersion (0.05 mg/mL) over time. S-7

Figure S11. a) UV-Vis spectrum of 4-amminosalycilic acid (red-line) and GO-SA **5** (black line). b) FT-IR of GO-SA **5**. c) TGA analysis in air of GO **1** (red line) and GO-SA **5** (black line). S-8

Table S1. Elemental analysis data related to mechanochemical synthesis of **5** performed on 4 different samples. S-8

Figure S12. QCM-D frequency shift (Δf) for all the overtones as a function of time during the formation of the adlayer: A) GO (**1**), B) r-GO (**2**), C) GO-SA (**5**), D) r-GO-SA (**4**). Samples' injection (1) was followed by milliQ water rinsing (2). Then, a ECE B and sodium perborate-based detergent was flushed into the measuring chamber (3), followed by a second rinsing step with milliQ water (4). S-9

Figure S13. Pictures of cotton fibers embedded with a) r-GO **2** (with SDC); b) r-GO-SA **4** (with SDC)**;** c) r-GO-SA **4** (without SDC)**;** d) GO **1**; e) GO-SA **5;** S-9

Figure S14: Raman spectra of pure GO and r-GO powders S-10

Figure S15: Raman map of the integrated G line intensity of graphene (1453-1669 cm^{-1}) over the surface of the cotton fabric, treated with: a) **1** (sample fabric+1); b) **2** (sample fabric+2). S-10

Figure S16: Raman map of the integrated G line intensity of graphene (1453-1669 cm^{-1}) over the surface of the fabric, treated with **4** S-10

Figure S17: Structure of sodium deoxycholate (SDC) S-11

Table S2 ....S-11

Synthesis of **2** (rGO)

Without sodium deoxycholate (SDC)

100 mg of **1** (spray dry powder) were dispersed in 400 mL of MilliQ water (0.25 mg/mL), then the same procedure described in the main text has been employed. UV-Vis: λmax = 270 nm (Figure S3a). FT-IR: 3425, 1552, 1519, 1404 and 1290 cm^{-1}(Figure S3b). Elemental analysis: C 45.50%, H 0.98 %, N 0.78 %

Using **1** (GO) water dispersion (0.4 % w/w)

12.5 mL of aqueous dispersion of **1** (4.0 mg/mL, 50 mg) were diluted to 50 mL with milliQ water. Then, SDC (200 mg) was added and the pH was adjusted at 8 with solid Na_2CO_3. The dispersion was treated in ultrasonic bath (1h, 59 Hz.). Then, 128 µL of hydrazine solution (30% in water) were added and the mixture was stirred at 80°C for 12 h. The crude mixture was used for further reaction without purification. UV-Vis: λmax = 270 nm (Figure S4a). FT-IR: 3739, 3256,1648, 1553, 1400, 1163, 1036.6, 831 and 697 cm-1 (Figure S4b). Elemental analysis: C 45.12 %, H 0.81 %, N 0.54 %

See Figures S3 and S4

Synthesis of **4** (r-GO-SA)

Without sodium deoxycholate (SDC)

The functionalization of r-GO **2** without surfactant was carried out accordingly to the procedure described in the main text. The diazonium salt was prepared using 5-fold excess in weight of **3** compared to r-GO **2** (0.2 mg/mL water dispersion). UV-Vis λmax= 210 and 270 nm (Figure S8a).

Loading of salicylic acid based on TGA under nitrogen atmosphere 5.39 % w/w (Figure S6b). FT-IR: 3739, 3618, 3226, 3107, 1743, 1550, 1211 cm-1 (Figure S8b).

Synthesis of r-GO-SA **4** (from **2**, prepared using the 0.4% w/w water dispersion of 1 (GO)

$NaNO_2$ (385 mg, 5.6 mmol) and 4-aminosalicylic acid (250 mg, 1.6 mmol) were dissolved in 20.4 mL of a NaOH solution (0.25 % w/v in water). The mixture was added dropwise to an ice cooled HCl solution (0.1 M in water, 26.6 mL) under vigorous stirring. The resulting solution was transferred, dropwise, to a dispersion of **2**-SDC (50 mg, 1 mg/mL) kept at pH 6 and at 0 °C under stirring. The final dispersion was sonicated for 1h (59 Hz) and then stirred at 80°C for 12 h. The reaction mixture was filtered (polycarbonate filters, 0.4 µm) and the filtrate was dispersed in water (1.0 mg/mL) and dialyzed vs water for four days to give 50 mg of pure r-GO-SA **4**. UV-Vis λmax= 270 and 211 nm (Figure S7).

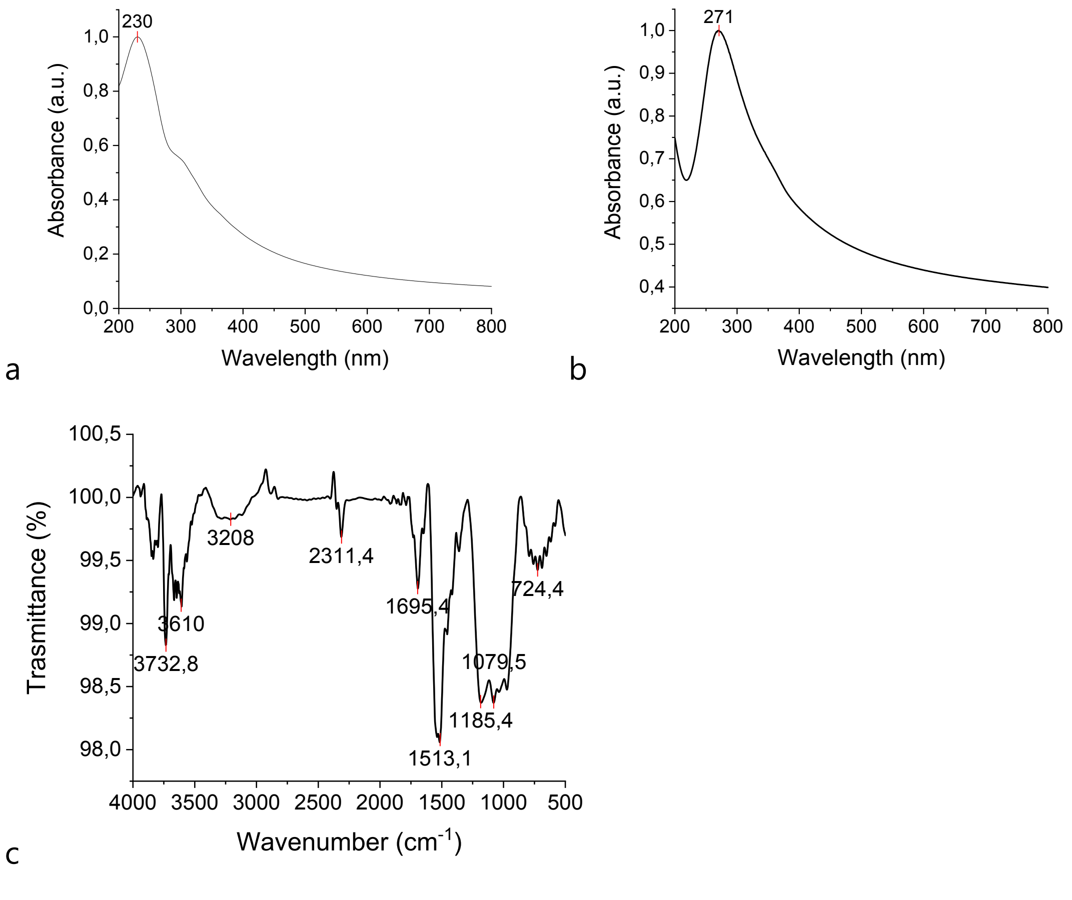

Figure S1. UV-Vis spectrum of a) GO (0.05 mg/mL in H_2O); b) r-GO **2** (0.05 mg/mL in H_2O) prepared from GO powder (λ_{max} = 271 nm) with SDC. c) FT-IR spectrum of r-GO **2,** KBr tablet prepared from GO powder.

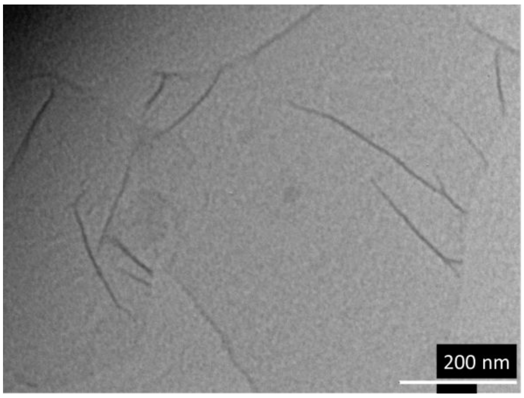

Figure S2. TEM image of r-GO **2** prepared from GO powder.

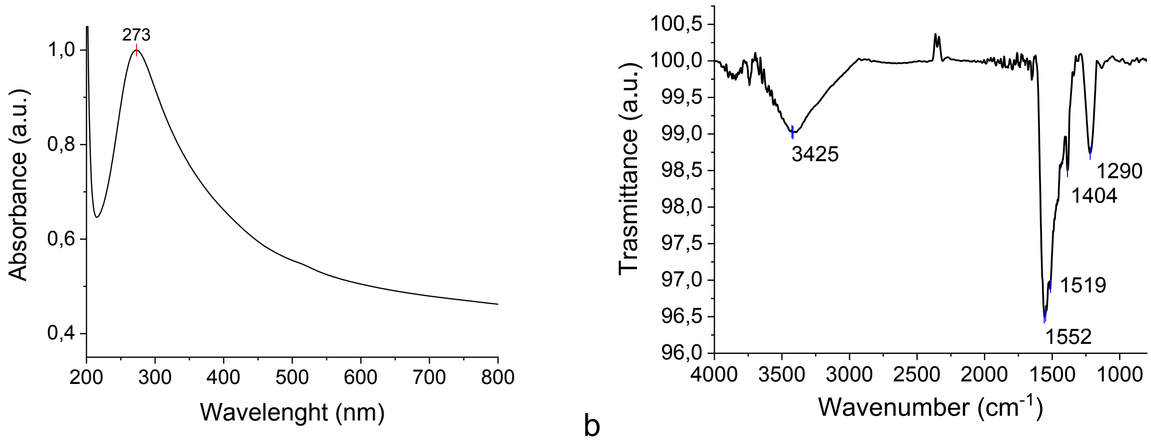

a b

Figure S3. A) UV-Vis spectrum of rGO **2** (0.05 mg/mL in H_2O) prepared from GO powder without SDC (λ_{max} = 273 nm). B) FT-IR spectrum of r-GO **2** KBr tablet prepared from GO powder without SDC.

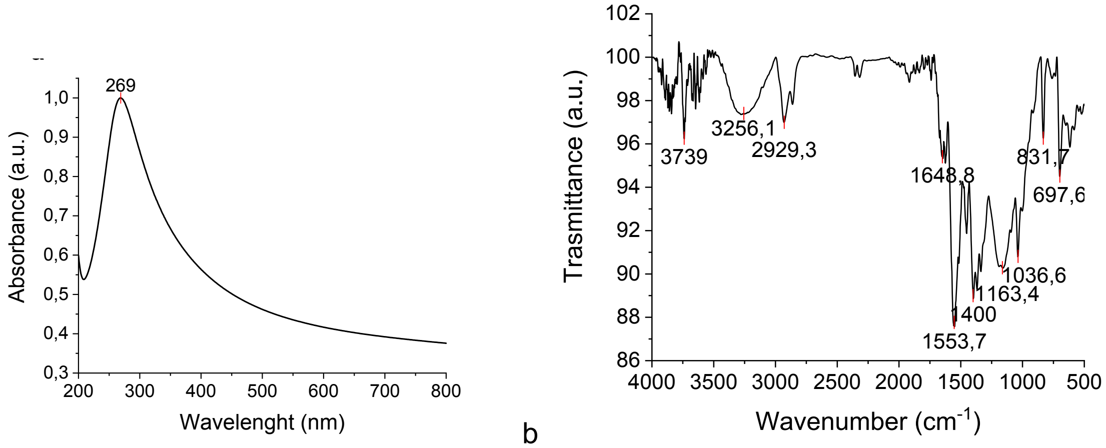

a b

Figure S4. A) UV-Vis spectrum of r-GO **2** (0.05mg/mL in H_2O) prepared from GO solution (0.4 % wt water GO). (λ_{max} = 269 nm). B) FT-IR spectrum of r-GO **2** KBr tablet prepared from GO solution (0.4 % wt water GO).

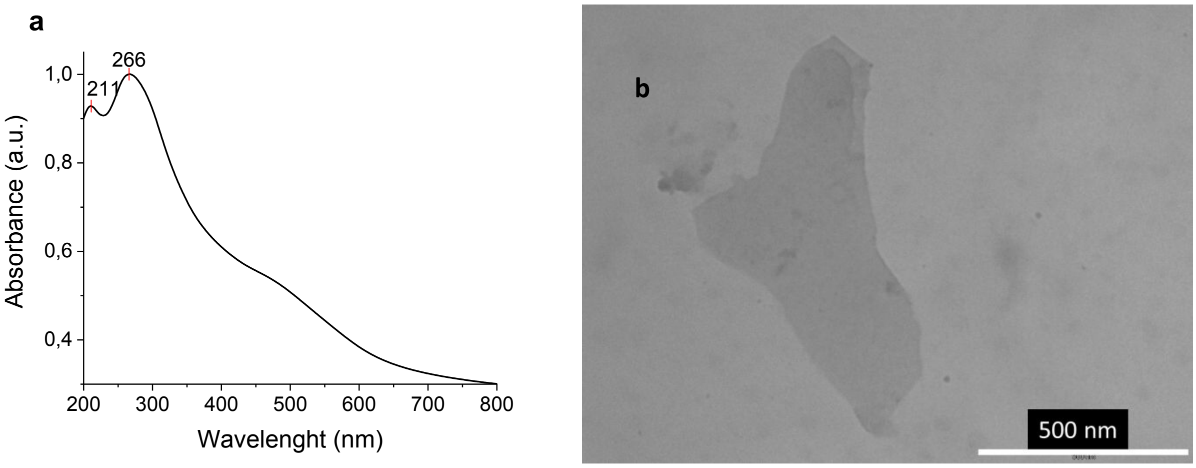

Figure S5. A) UV-Vis spectrum of r-GO-SA **4** (0.05mg/mL in H_2O); λ_{max} = 269, 211 nm b) TEM image of r-GO-SA **4**.

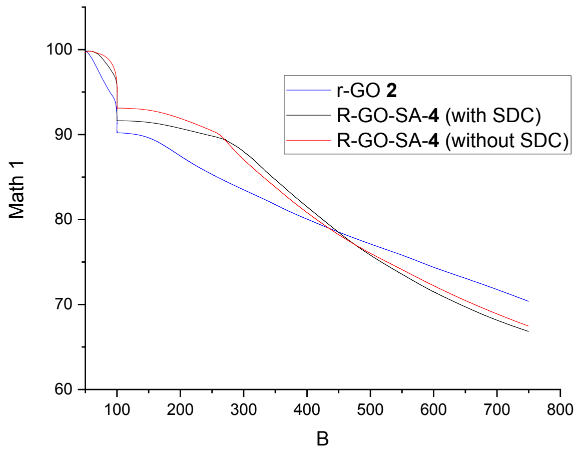

Figure S6. TGA analysis under nitrogen atmosphere of r-GO-SA **4**: a) with SDC (r-GO-SA **4 a**) (0.34 mmol/g, 4.70 % w/w) and b) without SDC (r-GO-SA **4 b**) (0.39 mmol/g, 5.39 % w/w). The weight loss calculated between 150 and 600 °C.

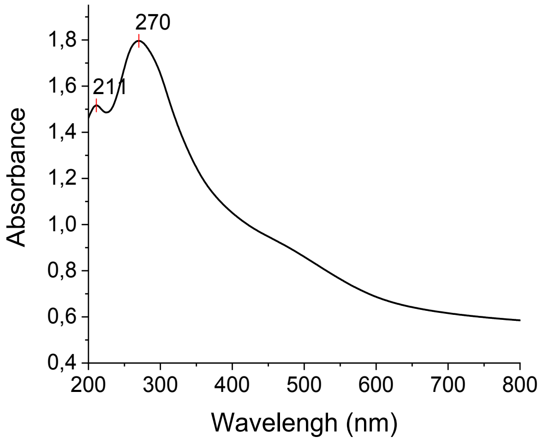

Figure S7. UV-Vis spectrum of r-GO-SA **4**

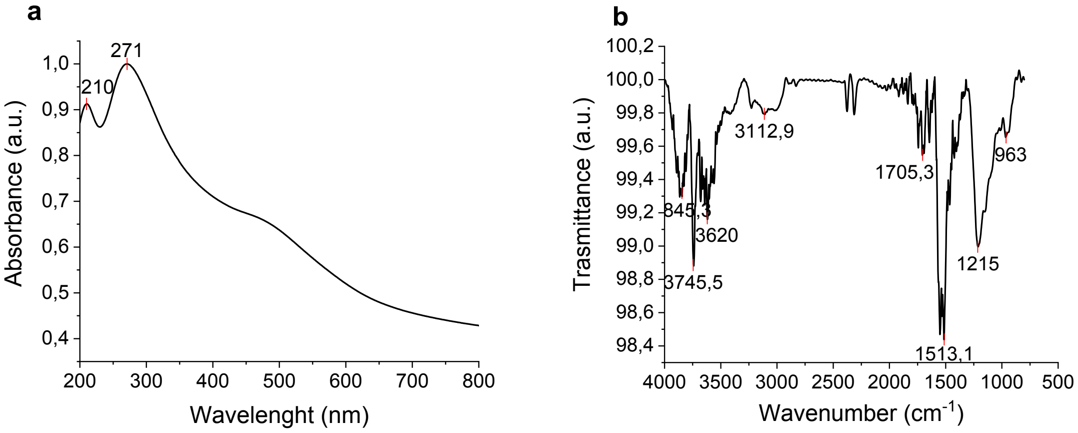

Figure S8. a) UV-Vis spectrum of r-GO-SA **4** (0.05mg/mL in H_2O); λ_{max} = 269, 210 nm. b) FT-IR spectrum of r-GO-SA **4** KBr tablet.

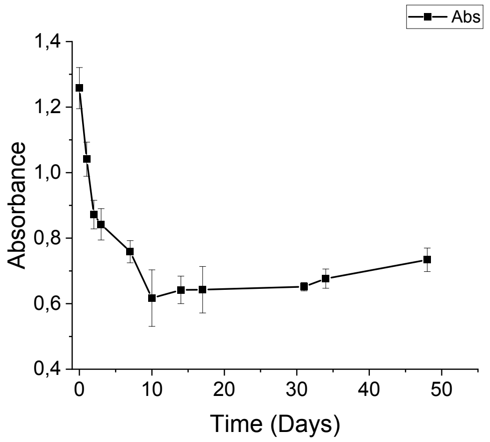

Figure S9. a) Intensity of UV-Vis spectrum of r-GO-SA **4** dispersion (0.05 mg/mL) measured at 270 nm over time.

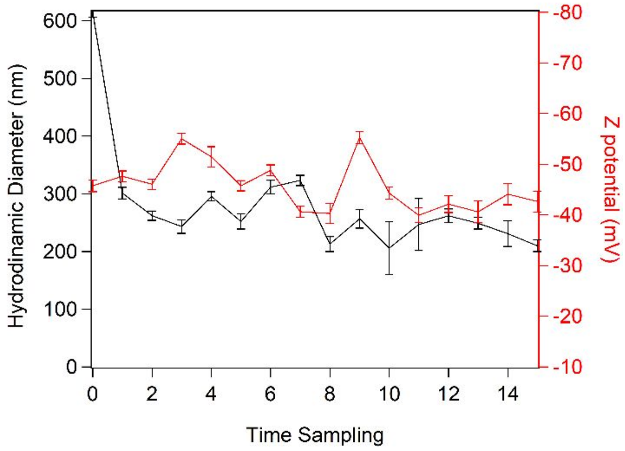

Figure S10. Hydrodynamic size of r-GO-SA **4** dispersion (0.05 mg/mL) over time.

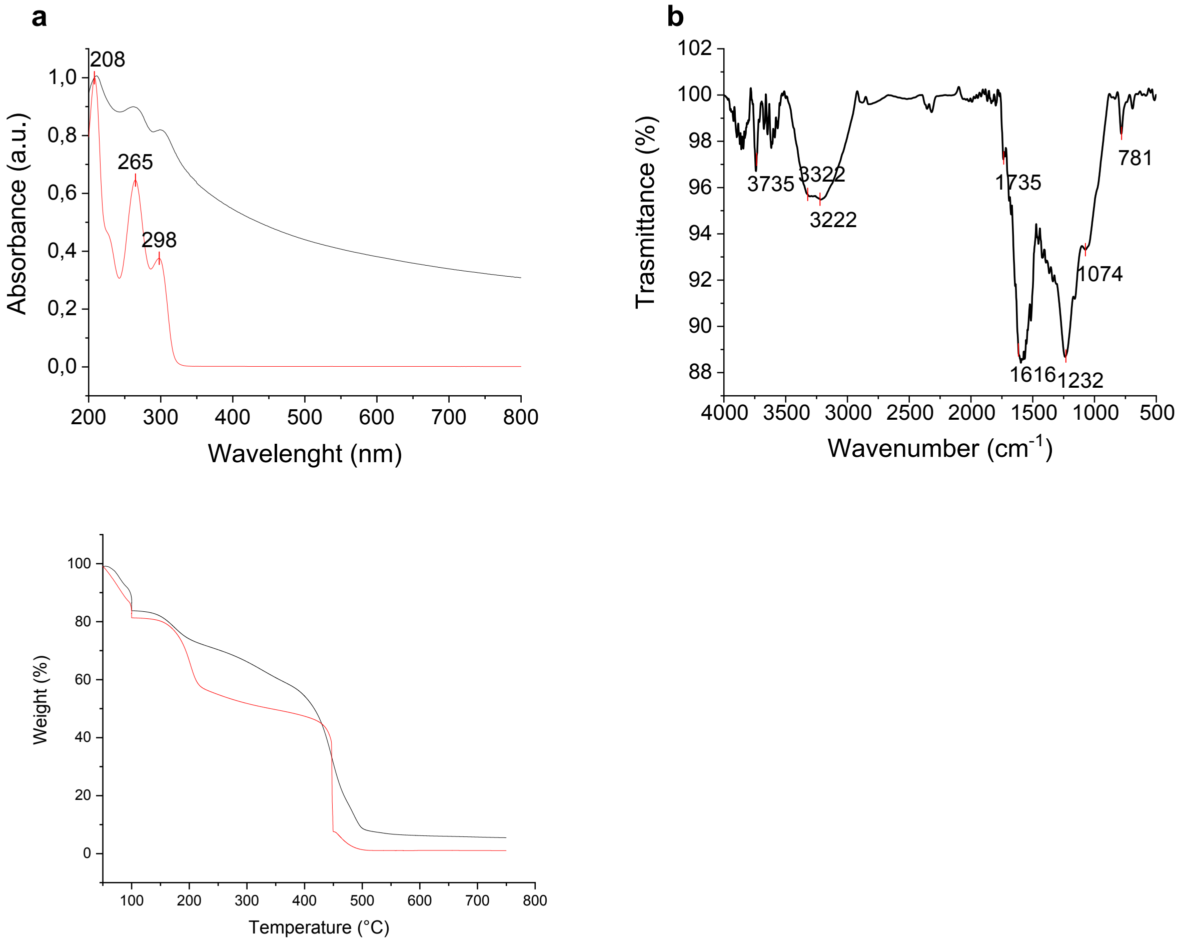

c

Figure S11. a) UV-Vis spectrum of 4-amminosalycilic acid (red-line) and GO-SA **5** (black line). b) FT-IR of GO-SA **5**. c) TGA analysis in air of GO **1** (red line) and GO-SA **5** (black line).

Table S1. Elemental analysis data related to mechanochemical synthesis of **5** performed on 4 different samples.

Sample	Ratio 4-ASA/GO	N%	C%	H%	Loading mmol/g
a	1:1	2,07	42.60	2.80	1,47
b	1:1	1,56	43,55	1.92	1,11
c	1:1	1,31	42,40	2,40	0,93
d	1:1	1,42	42,80	2,73	1,01

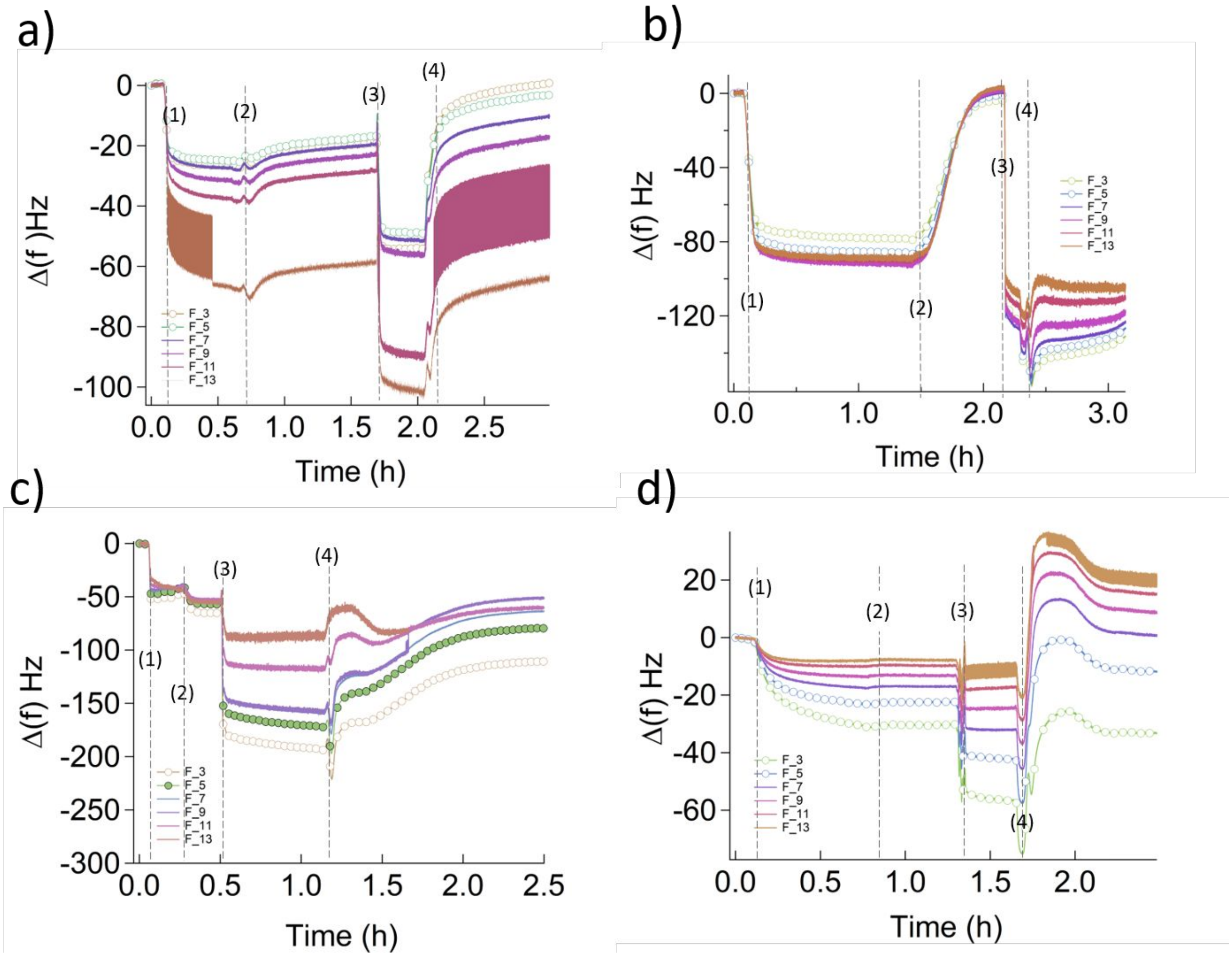

Figure S12. QCM-D frequency shift (Δf) for all the overtones as a function of time during the formation of the adlayer: A) GO (**1**), B) r-GO (**2**), C) GO-SA (**5**), D) r-GO-SA (**4**). Samples' injection (1) was followed by milliQ water rinsing (2). Then, a ECE B and sodium perborate-based detergent was flushed into the measuring chamber (3), followed by a second rinsing step with milliQ water (4).

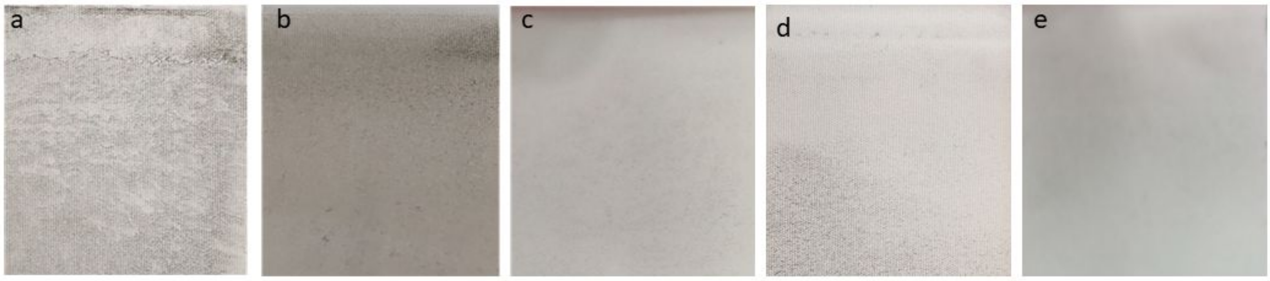

Figure S13. Pictures of cotton fibers embedded with a) r-GO **2** (with SDC); b) r-GO-SA **4** (with SDC)**;** c) r-GO-SA **4** (without SDC)**;** d) GO **1**; e) GO-SA **5;**

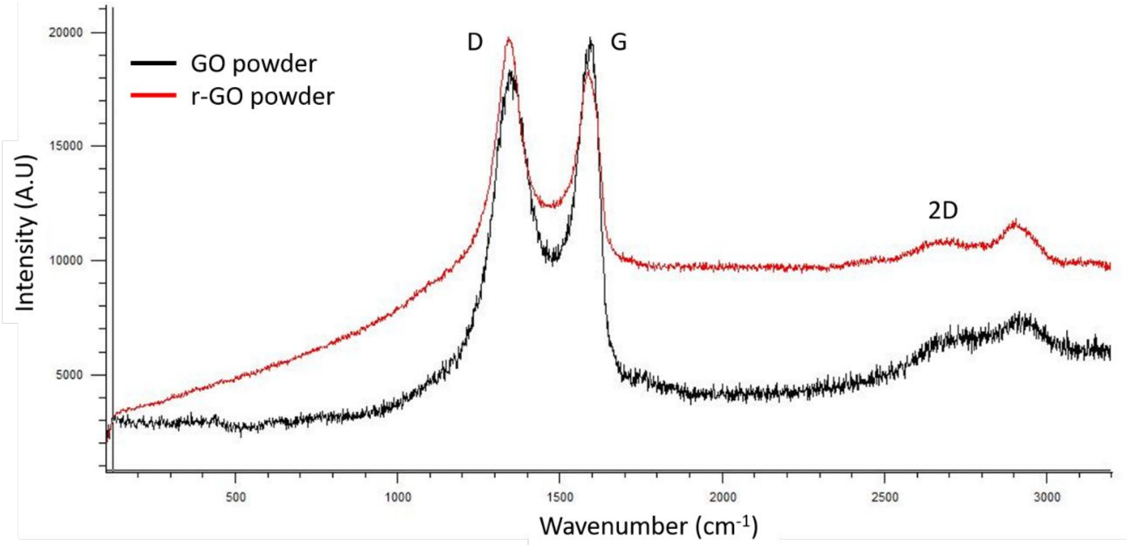

Figure S14: Raman spectra of pure GO and r-GO powders

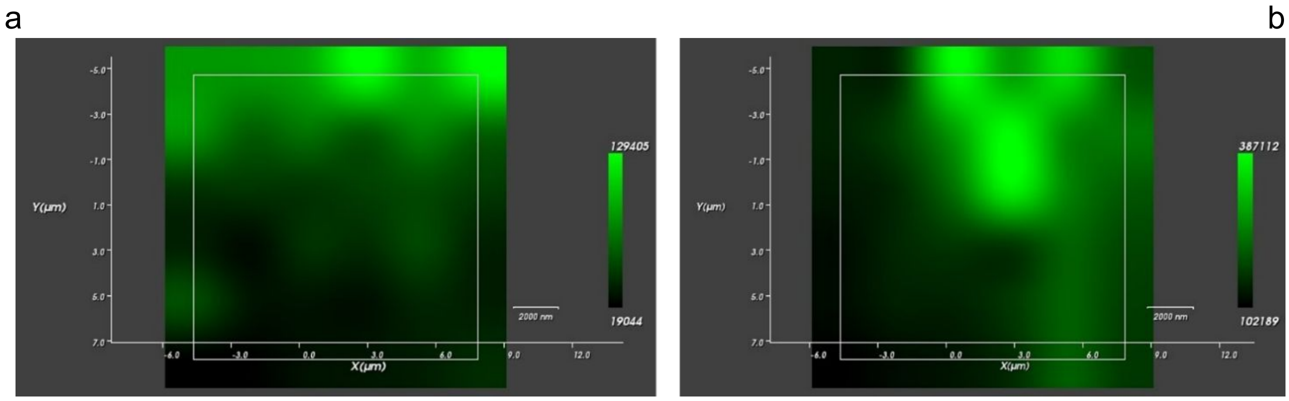

Figure S15: Raman map of the integrated G line intensity of graphene (1453-1669 cm^{-1}) over the surface of the cotton fabric, treated with: a) **1** (sample fabric+1); b) **2** (sample fabric+2).

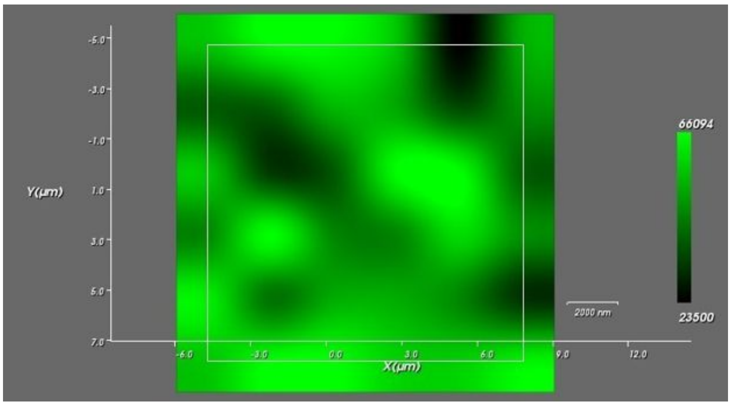

Figure S16: Raman map of the integrated G line intensity of graphene (1453-1669 cm^{-1}) over the surface of the fabric, treated with **4**

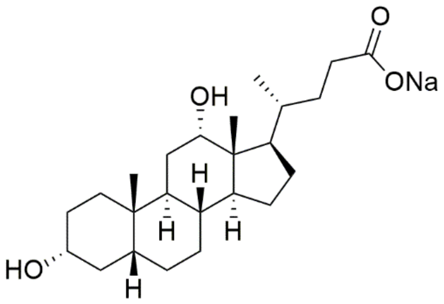

Figure S17: Structure of sodium deoxycholate (SDC)

Table S2

Sample	MCC (μg/mL)		
	K. pneumoniae	*S. aureus*	*C. albicans*
rGO **2**	128	2	>128
rGO-SA **4**	64	32	64
GO **1**	128	32	128
GO-SA **5**	64	>128	128

MCC= Minimal Cytocidal Concentration,.Table 1 shows the cytocidal values of the compounds tested against the *K. pneumoniae*, *S. aureus and C. albicans* strains
